# Passive longitudinal weight and cardiopulmonary monitoring in the home bed

**DOI:** 10.1038/s41598-021-03105-1

**Published:** 2021-12-21

**Authors:** Nicholas Harrington, Quan M. Bui, Zhe Wei, Brandon Hernandez-Pacheco, Pamela N. DeYoung, Andrew Wassell, Bayan Duwaik, Akshay S. Desai, Deepak L. Bhatt, Parag Agnihotri, Robert L. Owens, Todd P. Coleman, Kevin R. King

**Affiliations:** 1 Department of Bioengineering, Jacobs School of Engineering, University of California San Diego, 9500 Gilman Dr. MC 0412, La Jolla, CA 92093 USA; 2 Division of Cardiovascular Medicine, Department of Medicine, University of California San Diego, La Jolla, CA 92093 USA; 3 Department of Pulmonary, Critical Care and Sleep Medicine, University of California San Diego, La Jolla, CA 92093 USA; 4 Division of Cardiovascular Medicine, Department of Medicine, Brigham and Women’s Hospital, Boston, MA 02115 USA; 5 Population Health Services Organization, University of California San Diego, La Jolla, CA 92093 USA

**Keywords:** Circulation, Respiration, Biomedical engineering, Diagnostic markers

## Abstract

Home health monitoring has the potential to improve outpatient management of chronic cardiopulmonary diseases such as heart failure. However, it is often limited by the need for adherence to self-measurement, charging and self-application of wearables, or usage of apps. Here, we describe a non-contact, adherence-independent sensor, that when placed beneath the legs of a patient’s home bed, longitudinally monitors total body weight, detailed respiratory signals, and ballistocardiograms for months, without requiring any active patient participation. Accompanying algorithms separate weight and respiratory signals when the bed is shared by a partner or a pet. Validation studies demonstrate quantitative equivalence to commercial sensors during overnight sleep studies. The feasibility of detecting obstructive and central apneas, cardiopulmonary coupling, and the hemodynamic consequences of non-sustained ventricular tachycardia is also established. Real-world durability is demonstrated by 3 months of in-home monitoring in an example patient with heart failure and ischemic cardiomyopathy as he recovers from coronary artery bypass grafting surgery. BedScales is the first sensor to measure adherence-independent total body weight as well as longitudinal cardiopulmonary physiology. As such, it has the potential to create a multidimensional picture of chronic disease, learn signatures of impending hospitalization, and enable optimization of care in the home.

## Introduction

Heart failure (HF) is among the most challenging chronic conditions to manage. It affects more than 6 million patients in the US and costs more than $30B per year, largely due to the high burden of inpatient care required to manage recurrent exacerbations^1^. Hospitalizations for HF are often preceded by fluid accumulation with congestion and associated shortness of breath. If identified early, HF exacerbations can often be managed in the outpatient setting with temporary intensification of diuretic therapy^2,3^. Unfortunately, early detection currently relies on patient self-recognition and self-reporting of symptoms, which is unreliable, particularly during the COVID-19 pandemic, when patients are reluctant to go to hospitals^4^.

Patients are advised to self-measure daily weights and vital signs and to report sudden changes, but success of these strategies is limited by the need for patient engagement because there are no adherence-independent weight measurement devices for the outpatient setting^5^. In clinical studies, lack of adherence to self-measurement combined with variability of HF decompensation trajectories has prevented identification of a one-size-fits-all threshold of weight change that can trigger interventions to improve outcomes compared with usual clinic-based care^6–10^. Wearables are similarly limited by the need for patient engagement to charge and utilize sensors and apps^11,12^. Implantable pulmonary artery pressure sensors overcome some of these limitations and demonstrated reductions in HF hospitalization rates in the CHAMPION trial; however, the technology requires an invasive procedure, is costly, and requires ongoing adherence to patient-initiated data collection and transmission^13–15^. Patients with implanted cardiac rhythm management devices can be monitored for worsening HF using multiparameter monitoring of cardiopulmonary metrics; however, many HF patients (particularly those with preserved ejection fraction) do not require implantable pacemakers and defibrillators^16^. Given the limitations of existing remote monitoring technologies, we developed a non-invasive, multiparameter measurement technology that passively collects total body weight and dynamic cardiopulmonary physiology in the home bed without requiring any active patient participation.

Passive home monitoring has been achieved by embedding sensors into a wide range of everyday objects, facilitating measurement of respiratory signals and ballistocardiograms (BCG, a mechanical cardiac signal correlated with stroke volume and contractility) from healthy individuals and patients with chronic diseases^17–21^. For example, a modified stand-upon weigh scale was shown to classify HF patients based on 30-s BCGs, but the short duration of monitoring and the requirement for patient-initiated self-measurement leave it vulnerable to poor patient adherence^22–24^. Respirations and BCGs are often measured by piezoelectric or electromechanical film sensors placed above or below mattresses or bed frames, or via bedside radiofrequency transmit-receivers^25–29^; however, because these sensors do not span the entire body and are primarily sensitive to high frequency dynamic signals, they are unable to measure total body weight^30^. Since it is not uncommon for patients to present to the emergency room with 20 lbs of fluid overload, and because that amount of fluid does not accumulate in 1–2 days but instead does so gradually over days to weeks, a non-contact adherence-independent weight sensor has potential to enable early intervention for fluid overload in HF.

We developed an under-the-bed mechanical sensing platform (“BedScales") that achieves adherence-independent noncontact longitudinal physiological monitoring of total body weight, high fidelity respiratory signals, and BCG-derived heart rates. We validated the technology by comparing BedScales to ground truth data from commercial bathroom scales, chest respirometers, nasal flow sensors, and electrocardiograms used in overnight clinical sleep studies. We also demonstrated the feasibility of detecting respiratory pathologies including tachypneas, central and obstructive sleep apneas, and periodic breathing; ventricular arrhythmias and their hemodynamic consequences. Finally, we demonstrate the real-world durability of the platform by showing data from an example patient with HF and ischemic cardiomyopathy during a 3-month recovery from coronary artery bypass grafting surgery. By eliminating the need for patient participation, BedScales has the potential to improve care of patients with difficult-to-manage chronic cardiopulmonary diseases such as heart failure.

## Results

### Design of a non-contact adherence-independent sensor for chronic disease monitoring

**Figure 1:**
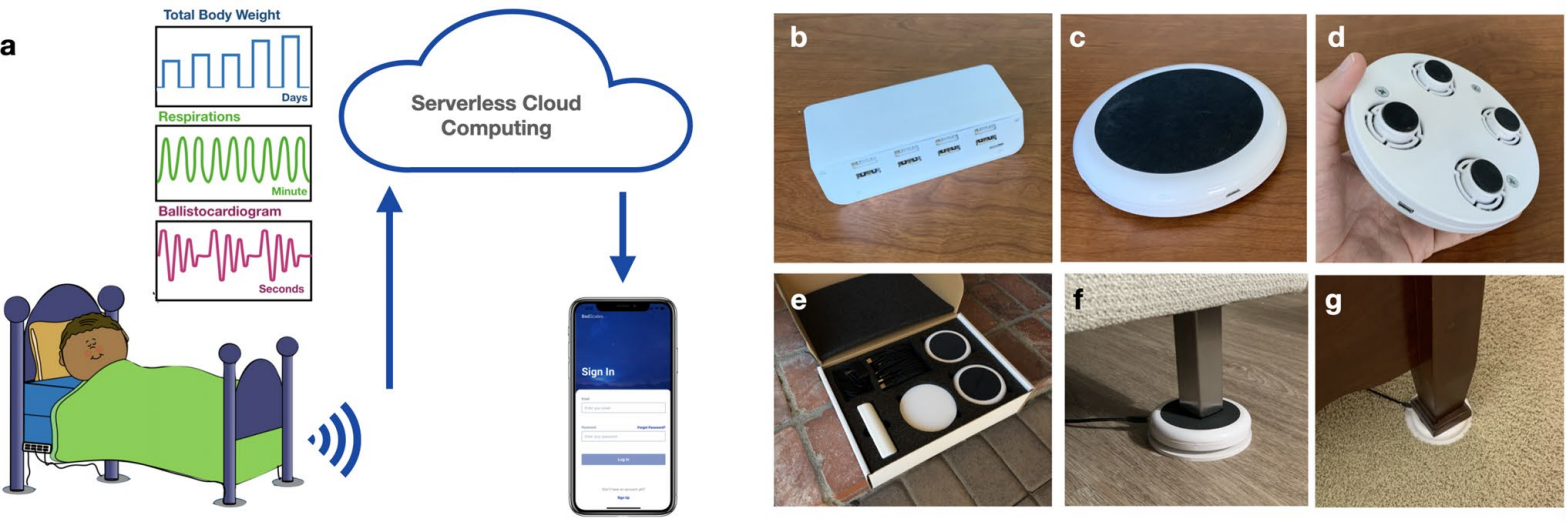
BedScales adherence-independent weight monitoring. (**a)** Illustration of the BedScales platform. Weight, respiratory, and cardiac physiology are automatically and continuously collected via non-contact sensors under the legs of the home bed, transmitted to the cloud, processed, and made available via web app, mobile app, or electronic medical record integration. (**b–g)** Images of the BedScales (**b)** communications hub, (**c)** top of sensor, (**d)** bottom of sensor, (**e)** packaging for shipment, and installed on (**f)** hard flooring and (**g)** carpet.

Roughly one third of life is spent asleep in bed. This is a unique setting in which cardiopulmonary physiology can be longitudinally measured without requiring correction for level of activity and without obscuration by musculoskeletal movements. To leverage this ideal diagnostic window, we designed, manufactured, and validated a non-contact fully adherence-independent home monitoring system called BedScales. It consists of low-profile force sensors beneath each leg of a conventional home bed, recliner, or couch, which measure and transmit 80 Hz sampled data to a cloud computing environment via WiFi, where physiological parameters and signals are quantified including total body weight, detailed respiratory waveforms, ballistocardiograms, and musculoskeletal movements, all without requiring any conscious patient participation, management of devices, engagement with apps, or self-application of wearables (Fig. 1a). Each non-contact low-profile sensor is comprised of force-sensing strain gauges connected to a custom signal conditioning circuit board that snap fits into the custom plastic injection-molded housing. The housing design includes a planar plastic spring mechanism, which focuses the entire load through the sensing elements and minimizes shunting of force via the surrounding plastic. The assembled device is outfitted with a rubber top to prevent lateral sliding and the feet are bonded to a rigid circular bottom plate to make the system performant even on carpeted bedrooms (Fig. 1b-g).

Digitized and amplified data from the individual force sensors is automatically transferred via micro-USB to a wall-powered central communications module, which in turn transmits it to a HIPAA-affiliate Amazon Web Services environment via WiFi. We selected a hardwired connection between the sensors and communications box to avoid the need for Bluetooth troubleshooting and to provide an indefinite power source that does not require battery changes. Once stored in a time-series database in the cloud, the data can be synchronously or asynchronously processed to create custom analytics, visualizations, and dashboards for permission-dependent sharing with patients, healthcare providers, or family and friends. In summary, the fully automated and adherence-independent install-once-use-indefinitely platform assumes nothing about patients’ technical literacy and does not require an accompanying smartphone or laptop computer. It is therefore accessible to patients who are socioeconomically disadvantaged, geographically distanced, or physically or cognitively impaired. Furthermore, because the devices are scalably manufactured, they are suitable for appropriately powered clinical studies.

### Passive weight monitoring

**Figure 2:**
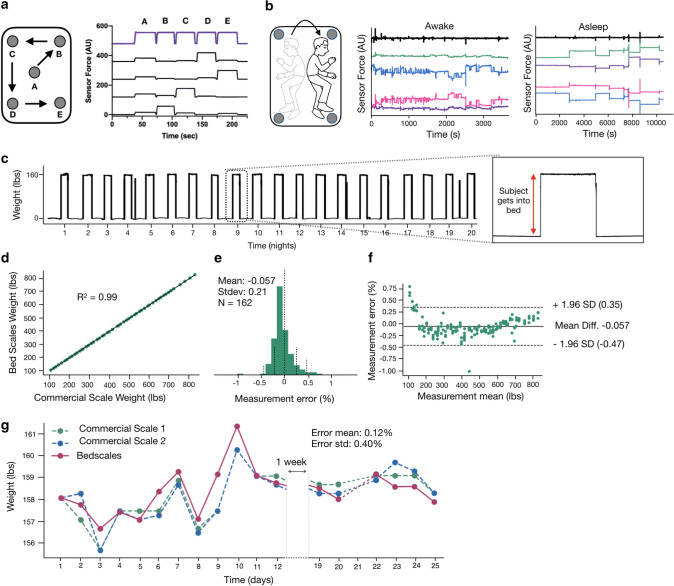
Non-contact adherence-independent longitudinal weight monitoring. **(a)** Measurement of individual (black) and summed (purple) sensor loads during movement of a 25 lbs weight from positions A through E (middle to the four corners of the bed). (**b)** Cartoon illustrating a sleeping subject redistributing total body weight during episodic movements in bed. Individual (colored) and summed (black) sensor loads are shown while a subject is awake in bed using a laptop with frequent redistributions of load (left) and during sleep with only episodic movements separated by long periods of lying still (right). (**c)** 3 weeks of the longitudinal sum of scales used to derive daily weights. Inset shows a single day of a person getting into and out of bed. (**d)** Correlation plot of BedScales versus commercial floor scale weights (R^2^ = 0.99, n = 162). (**e)** Histogram of measurement error, expressed as a percent of measured weight, comparing BedScales versus commercial floor scale (error mean -0.057% and standard deviation 0.21%). (**f)** Bland–Altman comparing BedScales versus commercial floor scale weights. (**g)** Comparison of daily BedScales weight measurements compared to two commercial bathroom scales. Pairwise error mean (0.12%) and standard deviation (0.40%) are shown.

Commercial weigh scales require that patients remember to self-initiate daily standing weight measurements, which limits their utility to engaged patients who can safely and stably stand on a home floor scale. Hospital beds measure patient weights when they are in bed using non-contact sensors, but they do so only at a single time point, leaving them vulnerable to unmeasured errors when blankets, pillows, books, and devices are added between the time of zeroing and measuring weight. In contrast, BedScales measures the weight of the bed and its contents continuously across time, which allows separate quantification of persons and objects based on the times that they are added or removed. For example, one can see the separate addition of a glass, increasing amounts of water, and recurrent placement and removal of a smartphone (Fig. S1). Weights are measured by summing the loads measured by the sensors beneath each bed leg. When an inanimate object of constant weight is moved to different locations on the bed to simulate a person changing positions in bed, the distribution of load amongst the sensors changes, but the total measured weight remains constant (Fig. 2a). Figure 2b illustrates the individual sensor measurements (color) and their sum (black) while a person is awake using a laptop compared to after they fall asleep. Note that movements become infrequent and episodic during sleep. In each case, despite movements and corresponding load redistributions, the total measured weight remains relatively constant. This is exemplified by the 3-week tracing of longitudinal adherence-independent home measurements shown in Fig. 2c.

Validation studies demonstrate that BedScales weight estimates are linearly correlated with commercial floor scales across clinically relevant weight ranges (R^2^ = 0.99, n = 162, spanning 100–800 lbs) (Fig. 2d-f). Errors between BedScales and commercial scales were clinically insignificant (mean error of −0.057% of total weight) (Fig. 2e,f). Additional characterization studies examining the lower limits of sensitivity demonstrated the ability to discern changes of 0.03 lbs and measure light-weight objects (e.g., smartphone) that are commonly placed onto the bed (Supplemental Fig. S1). Figure 2g shows a comparison of several weeks of daily weights measured by the BedScales compared to two commercial floor scales (each with a reported accuracy of ~ 0.2 lbs).

### Passive weight monitoring of multiple individuals sharing a bed

**Figure 3:**
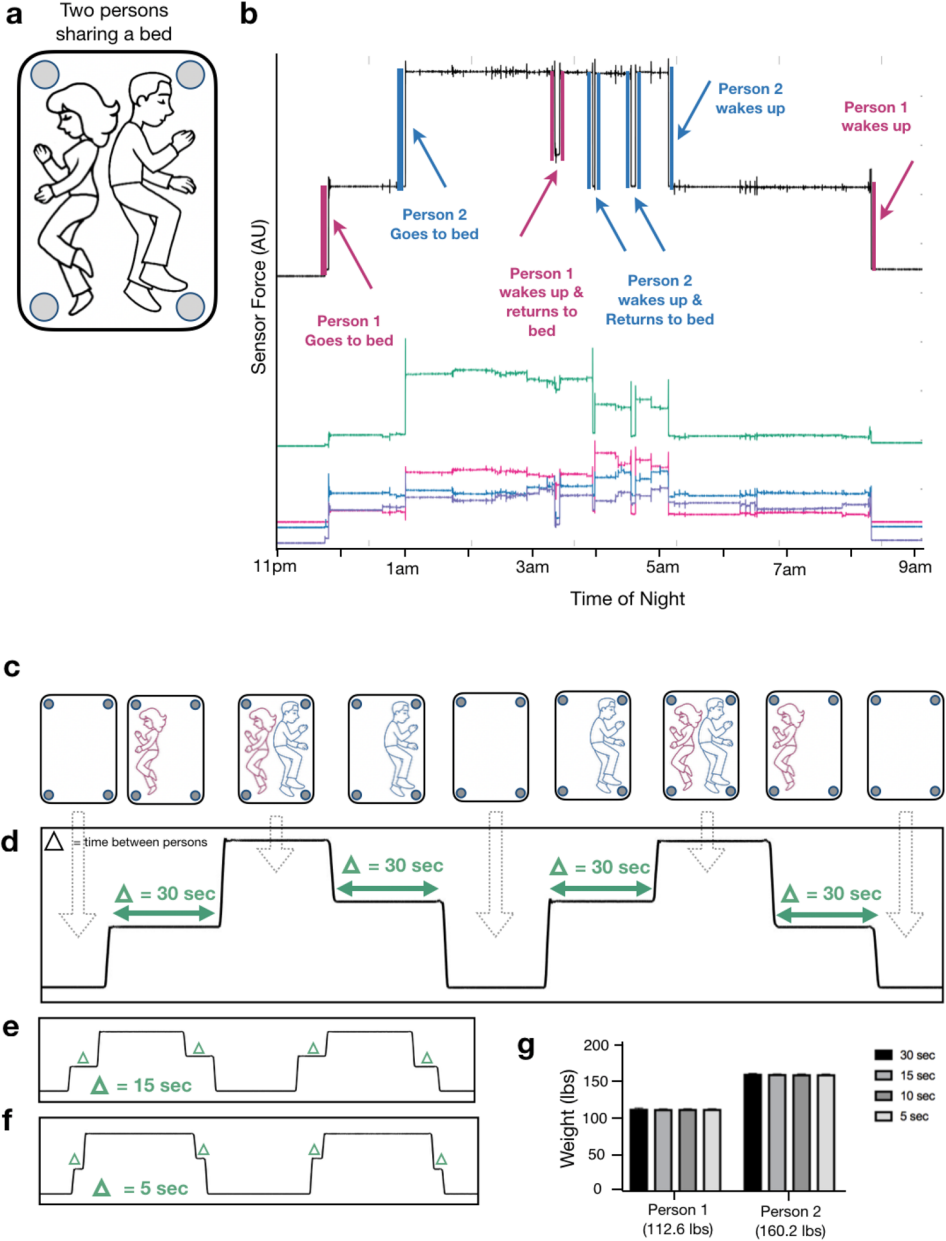
Non-contact adherence-independent multi-person weight monitoring. **(a)** Cartoon of partners sharing a bed. (**b)** Overnight measurements of two partners sharing a bed. Colored signals indicate individual sensor tracings. Black indicates the sum of sensors. Sudden weight changes due to each person getting into and out of the bed are color coded (blue and pink) and annotated. (**c)** Illustration of synchronicity protocol for measuring total body weights at progressively shorter time intervals. (Person = P) P1 On, P2 On, P1 Off, P2 Off, then repeated exchanging P1 and P2. (**d)** Corresponding total weight signal measured by the BedScales with a time interval of 30 s, (**e)** 15 s, and (**f)** 5 s. (**g)** Estimates of decoupled weights of the two individuals sharing a bed for each synchronicity time interval and corresponding weight measured by a commercial floor scale (mean + standard deviation).

Individuals often share the bed with a partner or pet (Fig. 3a); however, they rarely get into bed at precisely the same time. We reasoned those weights could be separately inferred based on the timing differences between their getting into and out of bed (Fig. 3b). To determine the minimum interval that would allow discrimination of two-person weights, we performed simultaneity tests in which two persons entered and exited the bed at successively decreasing time intervals (Fig. 3c). Even when the interval was reduced from 30 to 5 s, the two individuals were readily discriminated and weighed (Fig. 3d-g). Taken together, these data indicate that, compared with a conventional scale, BedScales can perform high-resolution total body weight measurements in a patient’s home bed, even if the bed is shared with a partner or a pet.

### Respiratory monitoring using non-contact bed sensors

**Figure 4:**
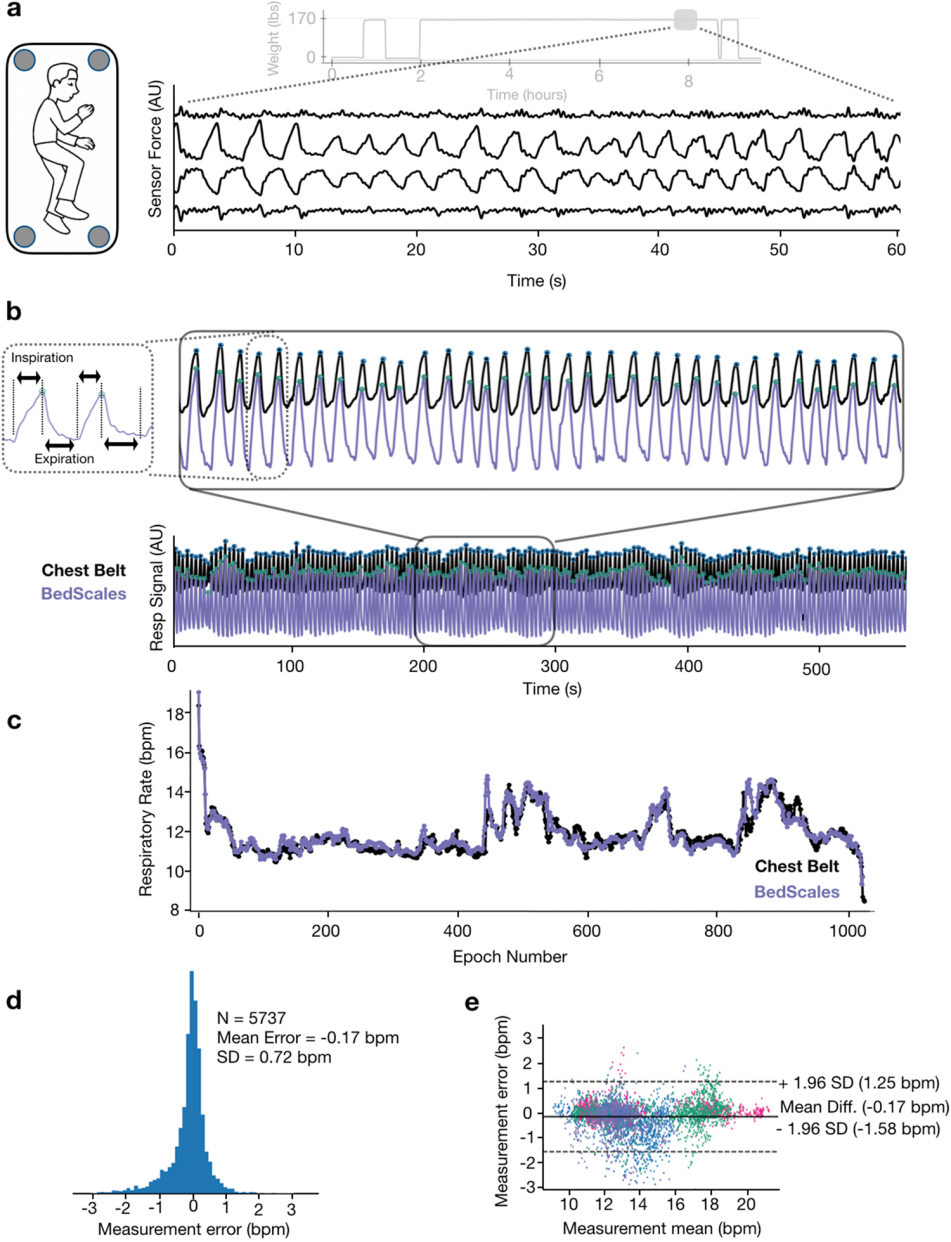
Respiratory monitoring using non-contact adherence-independent BedScales. (**a**) Raw respiratory signals from 4 scales in the middle of an overnight recording (light gray is the entire overnight weight signal). (**b**) Composite signal (purple) derived from linear combination of scales weighted by PCA-based eigenvalues compared to commercial respiratory chest belt (black) with peak finding annotation (green and blue dots respectively) across short (top) and long (bottom) time scales. Inset shows short inspiratory phase with rapid linear increase during inspiration followed by longer exponential decay during passive expiration. (**c)** Comparison of respiratory rates derived from BedScales (purple) and commercial respiratory chest belt (black) across one night (~ 1000 epochs). (**d)** Histogram of respiratory rate differences between BedScales and a commercial respiratory chest belt across 8 sleep study patients. X axis limits set at ± 1% quantile of error. (**e)** Bland–Altman plot comparing BedScales and chest belt respiratory rates. Y axis limits set at ± 1% quantile of error.

When a patient is asleep in bed, episodic musculoskeletal movements are separated by comparatively long movement-free intervals during which low variance physiological signals such as respirations and ballistocardiograms can be measured. This provides opportunities to perform adherence-independent longitudinal quantification of respiratory rate and detection of episodic tachypneas, apneas, and periodic breathing. BedScales respiratory signals arise from the dynamic redistributions of load that accompany chest wall movement during inspiration and expiration. To convert signals from multiple sensors into a single patient respiratory signal, we first performed bandpass frequency-dependent filtering of the individual sensor signals (cutoffs at 0.167 Hz and 1.5 Hz). We then used principal component analysis within a sliding window to calculate eigenvalues that, when multiplied by individual sensor signals and algebraically summed, create a single respiratory source signal for peak finding (Fig. 4a,b). The resulting signal enabled quantification of interbreath intervals and respiratory rates.

Respiratory signals exhibited expected contours with brisk linear upstrokes during inspiration followed by exponential decays during expiration (Fig. 4b), which are prolonged in obstructive diseases such as asthma or COPD^22–24^. To validate the measurements, we installed BedScales beneath the legs of a hospital bed during overnight sleep studies and compared the resulting signal to those obtained from the standard commercial chest belt respirometer (Fig. 4b,c). To facilitate comparisons, we estimated the respiratory rate every 30 s generating 5737 respiratory rate epochs from 8 patients and observed close quantitative agreement, with a clinically inconsequential mean error and standard deviation of −0.17 ± 0.72 bpm. This is shown longitudinally across time for a single patient (Fig. 4c) as a histogram of errors (Fig. 4d), and as a Bland–Altman plot (Fig. 4e).

### Respiratory monitoring of multiple individuals sharing a bed

**Figure 5:**
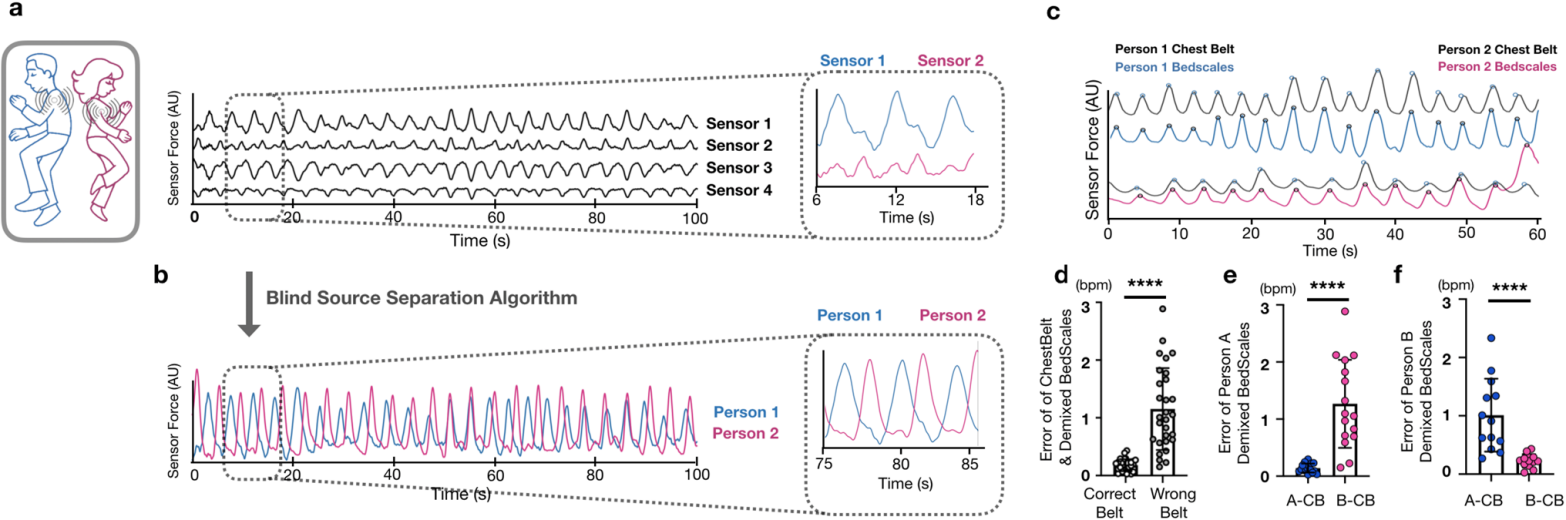
Strategy for separation of respiratory signals when two persons share the bed. **(a)** Cartoon illustrating how two persons sharing a bed are modeled as two respiratory point sources and raw signals from the 4 legs of the bed beneath two sleeping individuals. Inset shows 2 sensors each predominantly measuring one person with contaminating signal from the second person. (**b)** Demixed signals for person 1 (blue) and person 2 (pink) derived from the raw signals in A. (**c)** Validation experiment comparing demixed BedScales signals (blue and pink) with the corresponding ground truth chest belt signals (black). (**d)** Bar plot comparing peak location errors across both subjects between the demixed signal and the “correct chest belt “versus the error between the demixed signal and the “wrong chest belt.” (**e,f)** Bar plot quantifying the error between the BedScales separated signal from subject A **(e)** or B **(f)** and the chest belt on subject A (A-CB, left) or subject B (B-CB, right). Data are shown as mean ± standard deviation. ****P < 0.0001, Mann–Whitney test.

To enable demixing of respiratory signals from two persons who share a bed, we took advantage of the fact that between episodic movements, patients behave like respiratory point sources. In other words, the amplitude of their respiratory signal projects to the sensor beneath each bed leg with a relatively consistent magnitude (Supplemental Fig. S2). This allowed respiratory signals to be demixed using source separation mathematics as detailed in the methods^31^. One can see that when two individuals sleep in bed at the same time, their signals have distinct respiratory patterns that go in and out of phase (Fig. 5a). After respiratory source separation, one can qualitatively see that two typical respiratory signals emerge (Fig. 5b). To validate the strategy, we measured BedScales signals of two individuals sharing the bed while simultaneously recording ground-truth respiratory signals using commercial chest belts. The separated BedScales signals strongly correlated with those of the corresponding respiratory belt (Fig. 5c). Errors in respiratory peak timing of BedScales compared to the corresponding person’s chest belt (0.18 ± 0.08 s) were significantly less than comparisons to the opposite person’s belt (1.15 ± 0.71 s) (*P* < 0.0001) (Fig. 5d-f). These data establish the feasibility of using BedScales to monitor the respiratory status of multiple individuals sharing a bed.

### Heart rate monitoring using non-contact bed sensors

**Figure 6:**
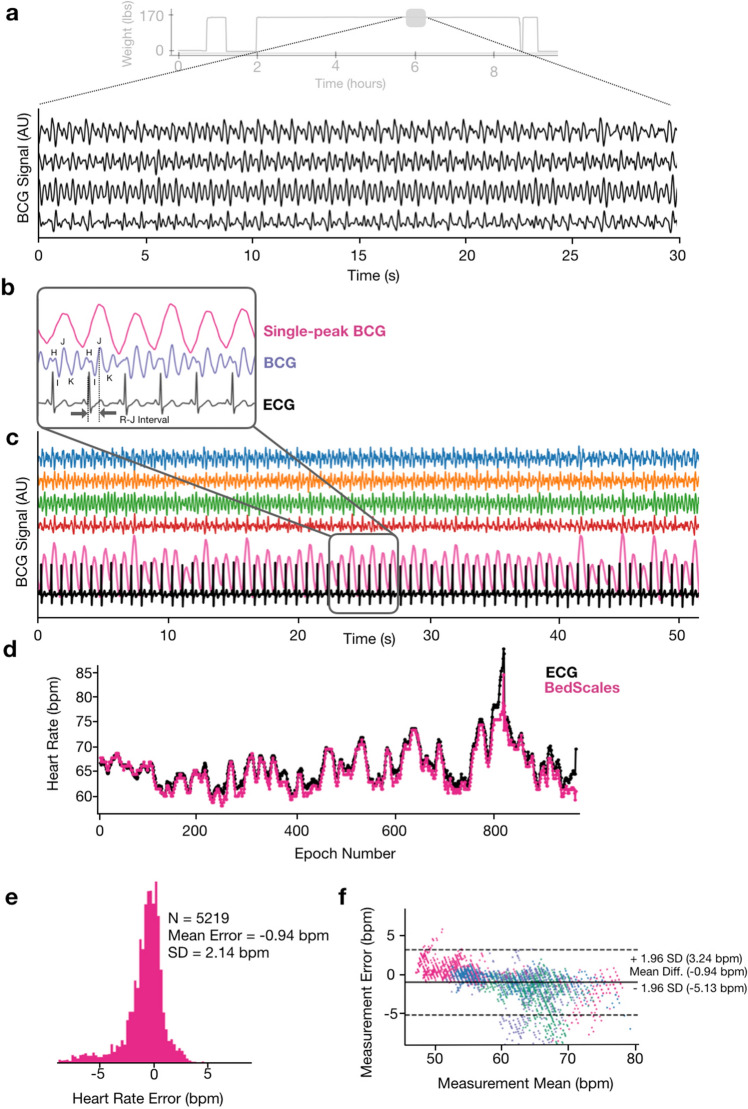
Adherence-independent longitudinal ballistocardiographic monitoring using BedScales. **(a)** BCG signals (black) from each of the 4 legs during an overnight recording, smoothed for display (light gray is the overnight weight signal). (**b)** Single-peak BCG (pink), BCG (purple) showing the labeled waveform (smoothed for display), and simultaneously recorded ECG signal (black). (**c)** Comparison of longitudinal BCG signals from the 4 individual scales (blue, orange, green, red), the single-peak BCG (pink), and the ECG (black). (**d)** Comparison of heart rates derived from BedScales (pink) and ECG (black) across one night (~ 900 epochs). (**e)** Histogram of heart rate differences derived from BedScales and from ECG across 8 sleep study patients (5219 epochs, mean error −0.94 bpm, standard deviation 2.14 bpm). X axis limits set at ± 1% quantile of error. (**f)** Bland–Altman plot comparing BedScales and ECG derived heart rates. Y axis limits set at ± 1% quantile of error.

The mechanical force of each heartbeat results in a characteristic signal known as the ballistocardiogram (BCG) with defined waves that follow each QRS complex of the electrocardiogram (ECG)^32^. Bandpass filtering of BedScales signals (5 Hz and 50 Hz cutoffs) revealed characteristic BCG morphologies from the individual scales (Fig. 6a,b). A single-peak BCG was derived by converting the raw BCG signal from each scale into an absolute measure of BCG energy (via a smoothed moving variance algorithm). The signals were then summed and filtered (bandpass, 1 Hz, 50 Hz) to create a final single-peak BCG metric, which was used for peak finding, heart rate estimation, and comparison to ground truth ECG-derived heart rates (Fig. 6c,d). This allowed longitudinal adherence-independent quantification of heart rate, regularity, and relative magnitudes of cardiac contractions. We validated the BedScales heart rate estimations by comparing to simultaneously recorded electrocardiograms. Heart rate estimates were made every 30 s, which generated 5219 epochs from eight patients. The data showed quantitative agreement with a clinically inconsequential mean error and standard deviation of −0.94 ± 2.14 bpm, which is displayed longitudinally across time for a single patient (Fig. 6d), as a histogram of errors (Fig. 6e), and as a Bland–Altman plot (Fig. 6f).

### Hemodynamic consequences of cardiopulmonary coupling and ventricular ectopy

**Figure 7:**
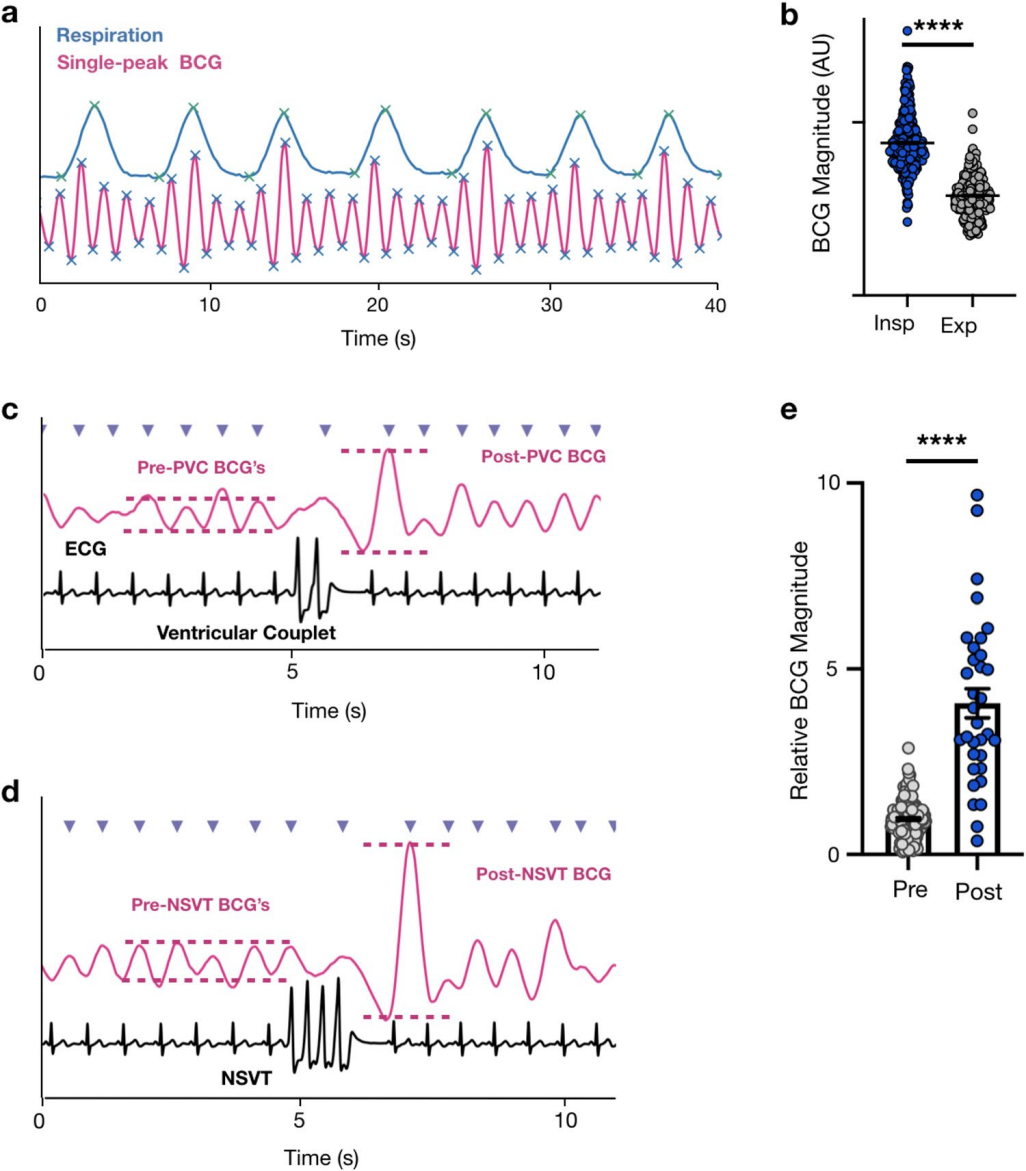
Cardiopulmonary coupling and hemodynamic consequences of arrhythmias. **(a)** Respiratory signal (blue) and single-peak BCG (pink) with peak and valley annotations (green and blue respectively) showing BCG magnitude variation with respiratory phase. (**b)** Bar plot comparing BCG magnitude of beats that began during inspiration (blue) compared to expiration (gray). (**c)** Single-peak BCG (pink) compared to ECG (black) with peak annotation (purple arrows) surrounding a ventricular couplet. Pink annotations highlight the BCG magnitude increase after the ventricular couplet. (**d)** Single-peak BCG (pink) compared to ECG (black) with peak annotation (purple arrows) during a region of non-sustained ventricular tachycardia (NSVT). Pink annotations highlight the BCG magnitude increase surrounding the NSVT. (**e)** Bar plot quantifying the relative magnitude of BCG beats occurring before ventricular couplets, triplets or NSVTs (gray) compared to the BCG beat immediately following the ectopy (blue). Data are shown as mean ± standard deviation. ****P < 0.0001, Mann–Whitney test.

Cardiopulmonary coupling arises when changes in intrathoracic pressure alter venous return, ventricular preload, and stroke volume. Indeed, we identified many regions where the magnitude of the inspiratory single-peak BCG amplitude was consistently and significantly greater than the expiratory amplitude (P < 0.0001) indicating respirophasic variation and cardiopulmonary coupling (Fig. 7a,b)^33,34^. We identified one sleep study patient with a high burden of ventricular ectopy, including premature ventricular contractions, ventricular couplets, triplets, and non-sustained ventricular tachycardia (NSVT) (Fig. 7c,d). Premature beats are often mechanically less productive than normal contractions due to short diastolic filling times. Meanwhile, post-premature beats are often mechanically stronger than a typical beat due to prolonged diastolic filling time. By quantifying the ratio of post-ectopy BCG magnitude compared to the preceding beats we found that BedScales can capture this well-known hemodynamic consequence of ventricular ectopy (Fig. 7c-e). Together these data demonstrate the feasibility of using BedScales BCGs to quantify regular and irregular rhythms as well as their hemodynamic consequences.

### Sleep study and apnea monitoring

**Figure 8:**
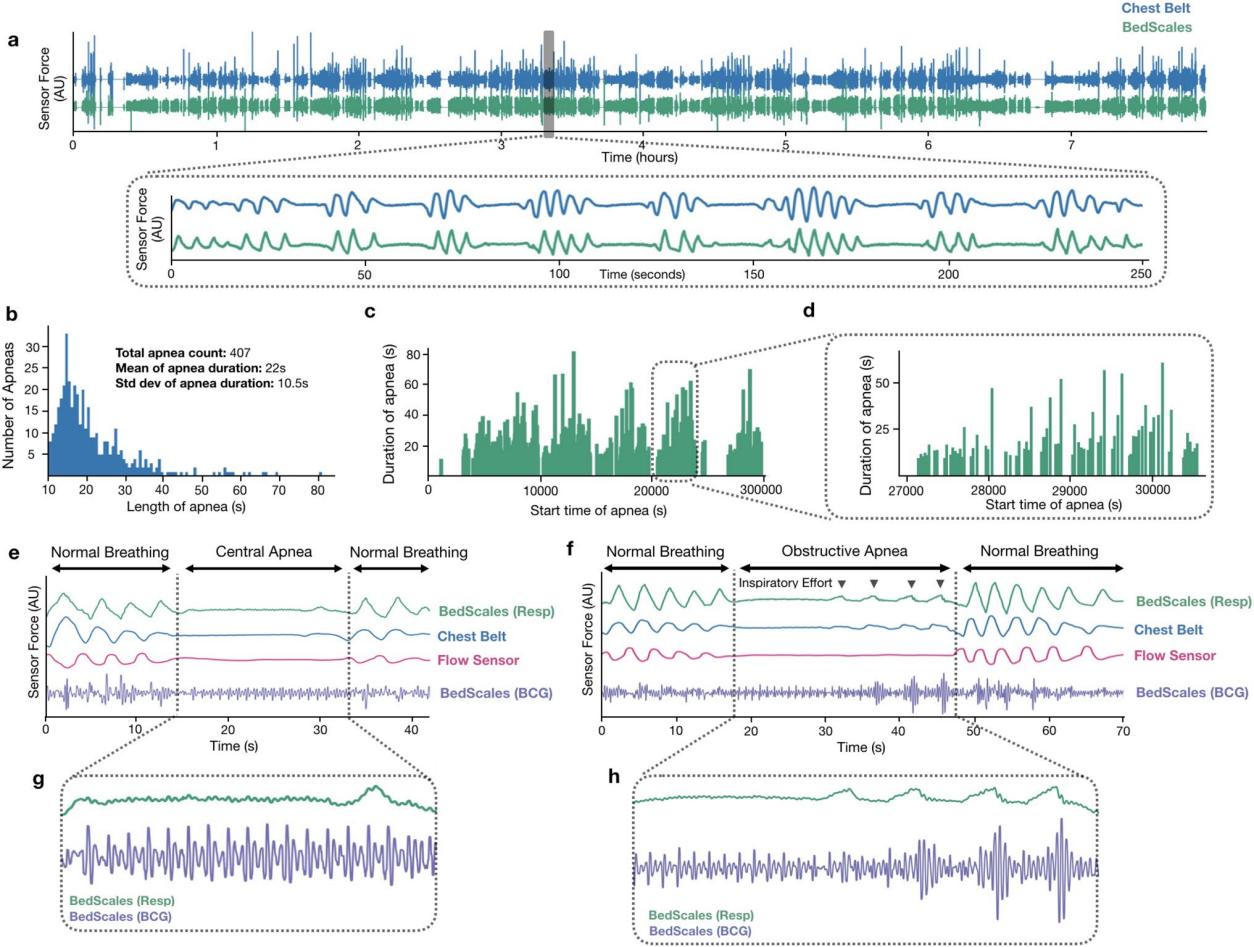
Example patient with mixed obstructive and central sleep apnea during simultaneous sleep study and BedScales monitoring. **(a)** BedScales non-contact respiratory signal (green) compared to chest respiratory belt (blue) during overnight sleep study. Inset illustrates the overnight burden of apneas. (**b)** Histogram of all apneas. (**c)** Duration of apneas vs timing of apneas throughout the overnight study. (**d)** High temporal resolution from one of the five apnea clusters during the night. (**e)** Central sleep apnea episode and (**f)** obstructive sleep apnea episode comparing BedScales respiratory signal (green) with chest belt (blue), nasal flow sensor (pink) and BedScales BCG (purple). (**g,h)** Insets show detailed BedScales respiratory and BCG signals during a (**g**) central apnea (no respiratory effort) and (**h**) an obstructive apnea (low amplitude respiratory effort against a closed airway).

We next examined an overnight sleep study patient with sleep disordered breathing who had a high burden of central and obstructive sleep apneas (CSA and OSA) (Fig. 8a). OSA is characterized by anatomical airway obstruction despite ongoing respiratory effort, whereas CSA is characterized by repetitive cessation of respiratory air flow during sleep due to lack of ventilatory effort; both are common in patients with HF^35–37^. During the ~ 8-h sleep study, we longitudinally measured respiratory signals from the BedScales along with the commercial chest respiratory belt as well as the nasal pressure airflow monitor. After aligning data, we quantified regions with low variance indicating a lack of respiratory waveforms. Regions greater than 10 s were defined as apneas and their distribution is shown in Fig. 8b. The mean apnea duration was 22 ± 10.5 s and the maximum apnea duration was 81 s. The distribution of apneas was periodic with 5 apnea-dense clusters separated by apnea-free intervals (Fig. 8c). Within each apnea cluster we observed substructure during which the longest apneas were followed by the longest apnea-free periods (Fig. 8d). Close examination of the tracings demonstrated that BedScales could discriminate central apneas (Fig. 8e) and obstructive apneas (Fig. 8f) based on the absence or presence of low amplitude unproductive respiratory efforts respectively. Examination of simultaneous BCGs showed stable amplitude signals in the absence of respiratory effort followed by transient increases in BCG amplitude following the strong negative intrathoracic pressure (Fig. 8g,h)^38^. Taken together, these data demonstrate the feasibility of performing high fidelity BedScales monitoring of normal and pathologic respiratory dynamics and their hemodynamic consequences without the need for obtrusive adherence-dependent sensors.

### Long-term in-home monitoring of a patient with heart failure

**Figure 9:**
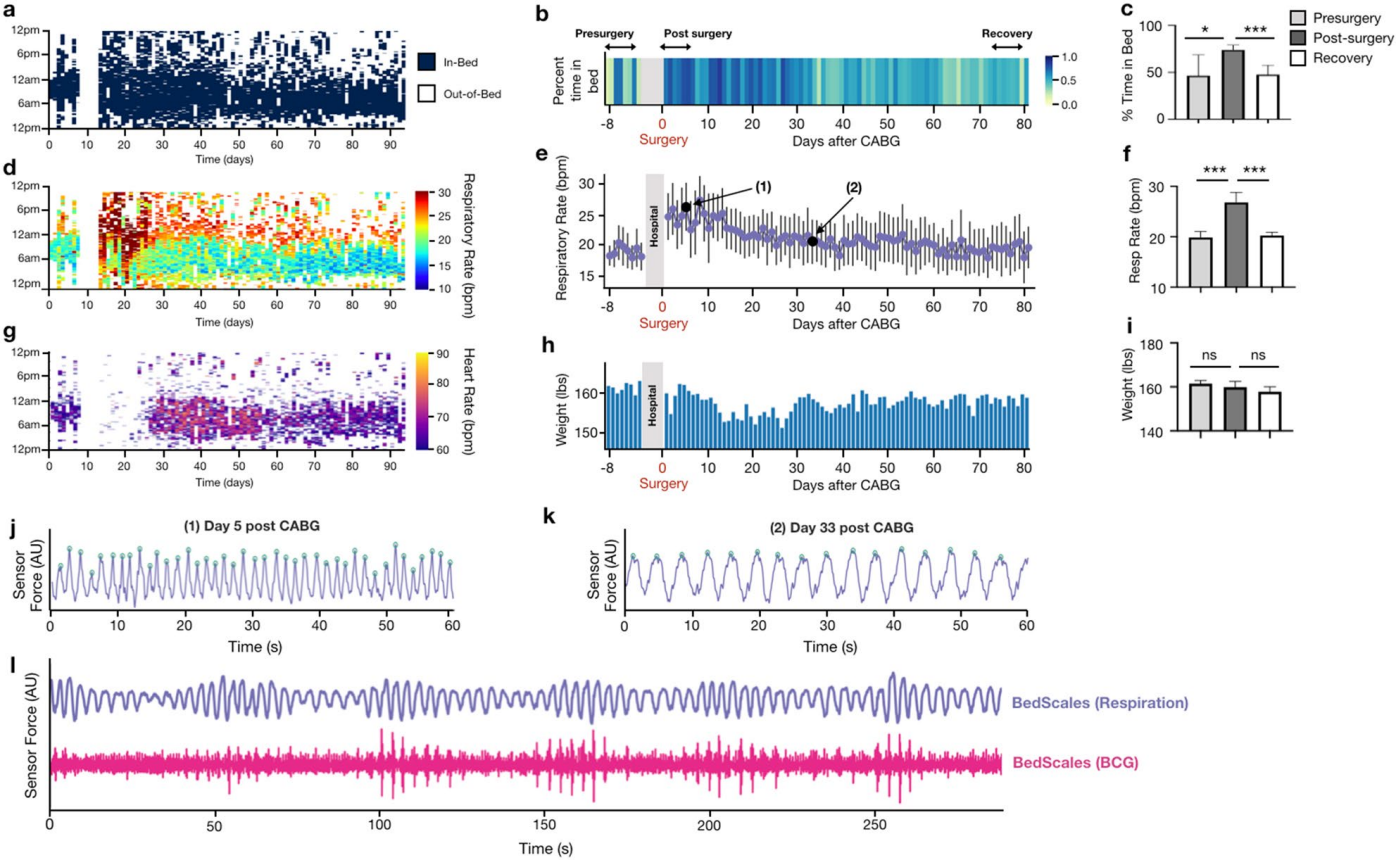
Adherence-independent longitudinal in-home monitoring of a heart failure patient using BedScales. (**a)** Heatmap showing binary in-bed and out-of-bed defined at each epoch. (**b)** Heatmap of daily percent time-in-bed (%TIB) from days 1–7 before surgery, and ~ 3 months after surgery. (**c)** Bar plot of %TIB comparing pre- post- and recovery from surgery. (**d)** Heatmap of respiratory rate (RR) during sleep as measured by the BedScales before and ~ 3 months after surgery. Values below minimum in color bar (10 bpm) not displayed. (**e)** Respiratory rate daily statistics before and after surgery. Example waveforms from days annotated (1) and (2) are shown in **(j–k)**. (**f)** Comparison of pre- post- and recovery from surgery RR. (**g)** Heatmap of heart rate (HR) during sleep as measured by the BedScales before and ~ 3 months after surgery. Values below minimum in color bar (60 bpm) not displayed. (**h)** Bar plot of total body weight (TBW) measured by the BedScales over the course of the 3-month in-home monitoring. (**i)** Comparison of pre- post- and remote-surgery TBW. (**j)** Example of tachypnea (> 40 bpm) in the early post-surgical period (black circle (1) in plot **e**). (**k)** Respiratory rate (16 bpm) one month after surgery (black circle (2) in plot **e**). (**l)** Periodic respirations suggestive on the spectrum of Cheyne-Stokes breathing and corresponding BCG measured by BedScales in this heart failure patient with a presurgical ejection fraction of 15%. Bar plots are shown as mean ± standard deviation. * *P* < 0.05, ** *P* < 0.01, ***P < 0.001, Mann–Whitney test.

To demonstrate the durability of BedScales during longitudinal monitoring in a real-world environment of a patient’s home, we show an example case of a ~ 60 year-old man who presented to the hospital with volume overload and newly discovered severely depressed ejection fraction due to ischemic cardiomyopathy. He was diuresed to euvolemia and discharged home with a plan to return several days later for coronary artery bypass grafting (CABG). At the time of discharge, BedScales were installed under his home recliner where he slept each night. We performed monitoring for several days prior to scheduled surgery and for 3 months during recovery. During that time, we quantified, every 30 s, (i) his in-bed and out-of-bed status as a binary quantity (Fig. 9a-c), (ii) his respiratory rate (RR) (Fig. 9d-f), (iii) his heart rate (HR) (Fig. 9g), and (iv) his weight (Fig. 9h-i). For compactness, cardiopulmonary data was plotted as a heatmap (Fig. 9d,g), such that the physiologic parameter defined every 30 s (e.g., RR, HR) was encoded as color and plotted with time-of-day on the y axis (24 h from noon-to-noon) and days on the x axis.

Mean and standard deviations of respiratory rate were 21.8 ± 2.5 and heart rate were 67.6 ± 2.4. The percent time spent in the recliner each day was on average 50% in the days prior to surgery and then significantly increased to ~ 80% for a month following surgery, before gradually declining to his baseline around the time he began attending cardiac rehab (Fig. 9a-c). His post-surgical RR during sleep was ~ 19 bpm, and when awake, his RR’s were punctuated by frequent episodes of extreme tachypnea (30–40 bpm), presumably due to sternal wound pain and chest wall changes that are expected after cardiac surgery (Fig. 9d,e,j). His respiratory rate gradually decreased over 2–3 weeks following surgery and stabilized near his baseline respiratory rate of ~ 16 bpm, consistent with previously reported respiratory rate recovery times following cardiac surgery (Fig. 9d,e,j,k)^39^. We quantified his RR for 7-days “pre-surgery”, 7-days immediately “post-surgery”, and 7-days at the end of “recovery” (Fig. 9f). Following a similar pattern to the respiratory rate, his heart rate also decreased to pre-surgical level over the course of 2 months (Fig. 9g). Although his weight fluctuated during the 3 months of monitoring, it did not show large excursions and he was felt to be euvolemic at clinic visits during the 3 months (Fig. 9h-i). Although his ventricular function modestly improved from 15 to 25%, it remained severely depressed based on echocardiography. For the first 2 weeks immediately following cardiac surgery, the quality of the BedScales BCG resulted in indeterminant heart rates, which we speculate is the result of dynamic intrathoracic changes that impact cardiac mechanical coupling to the chest wall. This improved within two weeks and the BedScales-derived heart rates gradually declined from the 70s to the 60s bpm. Consistent with his persistent ischemic cardiomyopathy was his high burden of periodic breathing (periodicity of > 30 s) along the spectrum of heart-failure-associated Cheyne-Stokes Breathing (Fig. 9l).

## Discussion

Here, we describe BedScales, the first non-contact adherence-independent total body weight sensor that also longitudinally quantifies cardiopulmonary dynamics throughout each night, as patients sleep in the comfort of their home beds. The sensor was made scalably manufacturable and two orders of magnitude less expensive than implantable medical devices intended for early detection of HF exacerbations. We validated its performance against commercial sensors in overnight sleep studies, demonstrated the feasibility of detecting pathologic features of sleep disordered breathing and the hemodynamic consequences of arrhythmias, and established the robustness of the platform by performing long-term monitoring in the uncontrolled environment of a patient’s home.

Future work will focus on longitudinal home monitoring of patients with chronic diseases such as heart failure, chronic kidney disease, and chronic liver disease, who are managed with diuretics in the outpatient setting and are at high risk of hospitalization. These patients are advised to self-measure daily weights using standing scales and to notify clinical care teams if a specified amount of weight is gained in a specified time so that diuretics can be titrated. Unfortunately, optimal weight change thresholds for intervention are unknown because longitudinal observational studies are not reported in detail and have been confounded by low adherence. BedScales offers the opportunity to overcome the adherence barrier. Beyond weight, BedScales can also measure nocturnal respiratory rate and heart rates without adherence. It remains unknown whether night-to-night variability in these biomarkers can improve early recognition of impending hospitalization and discrimination of volume overload from other respiratory diseases such as pneumonia, COPD, fibrotic lung disease, or cancer. Going forward, it will be important to perform prospective studies of well-defined patient cohorts to link BedScales longitudinal data to adjudicated clinical events.

There are several limitations to our work. BedScales must be placed under the legs of furniture, which typically requires that a person other than the patient lift each corner of the bed, position the sensors, and input the WiFi credentials. However, once the 10-min installation is complete, the sensor automatically monitors and transmits data for months at a time without any patient participation. Furniture compatibility represents another limitation. While BedScales are compatible with a wide range of recliners, couches, and other furniture with legs, they are not compatible with all beds, particularly those that are affixed to the ground or that cannot be supported by sensors at discrete locations. An additional limitation is the variable quality of BCGs measured during long-term in-home studies and the challenges of demixing BCGs from two persons. It is possible that an optimal home bed sensing solution will combine BedScales with bed-side radar or piezoelectric mattress sensor so that one can robustly perform adherence-independent longitudinal measurement of weights, respirations, and BCGs in the home.

In conclusion, BedScales offers a new platform for learning signatures of impending hospitalizations for heart failure and beyond. In a healthcare environment that is transitioning from fee-for-service to value-based care, BedScales has the potential to make outpatient chronic disease management a data-driven science and in doing so, achieve the triple aim of improving patient satisfaction, improving quality and access for populations, and reducing health care costs^40^.

## Methods

### Human subjects

All experiments were performed in accordance with relevant guidelines and regulations in accordance with UCSD Human Research Protections Program (HRPP) protocols IRB # 171480 and IRB # 180160. All experimental protocols were approved by UCSD HRPP IRB. Informed consent was obtained from all subjects.

### BedScales design and construction

Custom housing was designed using Solidworks (Waltham, MA). Tooling was machined and injection molded parts were manufactured by S. Pawlicki. 50 kg strain gauges were purchased from Sparkfun (Niwot, CO) or a comparable vendor. Custom circuit boards were designed in CircuitMaker (Altium) and included Hx711 integrated circuit technology (Avia Seminconductor) with a gain of 64 and a sampling frequency of ~ 80 Hz. The circuit board design was based on an Hx711 breakout board schematic, a load cell combinator schematic, and their corresponding Eagle files, all from Sparkfun (Niwot, CO). The circuit boards communicated via microUSB to a Raspberry Pi which received electrical power from the building wall outlet, which in turn powered each sensor. The transducers and custom circuit were snap fit into the housing which was secured with screws. Rubber tops were die-cut from rubber sheets and the scales were securely fixed to plastic plates using double-sided adhesive pads. The Raspberry Pi communicated data using the subject’s home WiFi and securely transmitted data to Amazon Web Services S3 buckets. All analyses were performed in Python and Matlab (Mathworks, Natick, MA).

### Weight measurement

Bed sensor weight validation was performed by using five healthy volunteers and two static weights in various permutations to span a large range of loads. Each person was measured on a commercial bathroom scale before lying on a bed with the BedScales sensor under each of its 4 legs. The 4 scales were calibrated together by fitting coefficients that minimized the variance when the same load was applied in different places. The final weights were calculated by subtracting the total load measured after and before each permutation of individuals and weights was placed on the bed (3 measurements were made of each permutation and these were averaged together). Two-point calibration that minimized the measurement error was then used to convert from arbitrary units (AU) to pounds (lbs). Longitudinal weight comparisons were made by installing BedScales under a home bed and comparing to self-measurement on two separate commercial floor scales at the beginning of each night of sleep. The limits of sensitivity were tested by placing the sensors beneath a 4-leg couch and placing an empty glass on a flat cutting board. At 20 s intervals, 15 mL (0.033lbs) aliquots of water were added. The glass was removed and replaced with and without water, and a smartphone was repeatedly added and removed.

### Respiratory measurement

BedScales respiratory signals were generated by frequency-dependent filtering with cutoffs of 0.167 Hz and 1.5 Hz. A single respiratory signal was derived by linearly combining the individual sensor respiratory signals weighted by PCA eigenvalues calculated for each 12.5 s window. A moving variance algorithm was used to isolate regions of steady physiology (regions < 10 s were rejected) and peak finding was performed on these regions. The respiratory rate was calculated using the median inter-peak interval during a 5-min moving window with a shift of 30 s. For validation, the BedScales respiratory signal and chest belt were subject to additional smoothing (moving mean, 0.5 s), windows were required to have no more than 45 s of unstable physiology, and regions with technical artifacts in the chest belt were excluded. Data was aligned and compared to a simultaneously recorded respiratory chest belt with respiratory rates quantified using the same method.

### Ballistocardiographic measurement

BCG signals from each scale were derived by frequency-dependent filtering (Butterworth) with cutoffs of 5 Hz and 50 Hz (lower cutoff set to 1 Hz during BCG amplitude analysis). These signals were then smoothed using a moving mean filter and moving variance filters. The resultant signals from each scale were then summed to create a composite signal, which was filtered using another frequency-dependent filter (Butterworth) with cutoffs of 1 Hz and 50 Hz. The resultant signal was a single peak measure of BCG. For in-home data, steady regions as defined by the respiratory signal were isolated and analyzed. For validation, a moving variance algorithm was used to isolate regions of steady physiology (regions < 5 s were rejected) and peak finding was performed on these regions. The heart rate was calculated using the median inter-peak interval during a 5-min moving window with a shift of 30 s. For validation, windows were required to have no more than 45 s of unstable physiology and a region with technical artifacts in the ECG was excluded. Data was aligned and compared to a simultaneously recorded ECG signal with heart rates quantified using the same method.

### 2-person weight demixing

The weights of persons sharing a bed were determined by measuring the calibrated sum of all sensors across time and extracting the large differential weight changes. The weight changes were then classified into two groups termed person 1 and person 2. Simultaneity tests were performed by instructing 2 persons to get into and out of bed at specified temporal intervals in the following sequence—Person 1 (IN), Person 2 (IN), Person 1 (OUT), Person 2 (OUT) and then repeated but exchanging Person 1 and 2. To explore the limits of simultaneity that would still permit decoupling of person weights, we systematically decreased the interval between Person 1 then 2 (or 2 then 1) getting into and out of bed and repeated the experiment for several time intervals (30 s, 15 s, 10 s, and 5 s), until the maneuver could not reach a steady position in the allotted interval.

### 2-person respiratory demixing

Demixing of respiratory signals obtained from two simultaneous sleepers was performed using a hidden Markov model. Mechanical respiratory sources were interpreted as latent signals that evolve in a stochastically continuous manner, according to a linear additive Gaussian model, mixed through a linear operation with additive sensor noise to give rise to the signals at the four detectors. Interpreting the linear operation as unknown, we used the Expectation–Maximization algorithm to obtain the maximum-likelihood estimate^41^. Given this estimate, the Kalman smoothing algorithm was used to extract the mechanical respiratory patterns of the two sources^42^. Validation was performed by simultaneously but independently measuring each respiratory signal using two respiratory belts (Vernier, Beaverton, OR). Interbreath intervals were compared by measuring the error between each demixed signal and each ground truth respiratory belt signal. For each individual, the absolute error between the putative demixed source signal and each respiratory belt signal was calculated and compared using a t test.

### Clinical sleep studies

BedScales were installed beneath the legs of a conventional hospital bed in the Clinical and Translational Research Institute where overnight sleep studies were conducted. As part of another ongoing study, subjects underwent standard in-laboratory polysomnography (PSG) with electroencephalogram (EEG), electro-oculogram, submental and leg electromyogram for sleep staging; nasal pressure and thermistor for airflow measurement; thoracic and abdominal piezoelectric bands for respiratory effort; arterial oxygen saturation monitoring at the finger; and electrocardiogram monitoring for safety. Patients slept supine. Sleep state, arousals, and respiratory events were scored by a registered sleep technologist according to standard American Academy of Sleep Medicine 2012 Recommended Criteria. Signals from the thoracic piezoelectric band and the BedScales were aligned using custom python scripts.

### In-home long-term studies and detection of hospitalizations and clinical events

BedScales were delivered to and installed under each patient’s bed (or recliner or couch). Signals were recorded locally and transmitted securely via the patient's WiFi to an Amazon Web Services S3 bucket which activated a cloud pipeline of analytics that extracted the physiologic parameters automatically. The daily average respiratory rate from each patient was derived by averaging the epochs (defined every 30 s) that had a physiologically reasonable bpm (bpm > 6 and bpm < 40). Values in the respiratory heatmap below 10 bpm were not displayed. The average heart rate from each patient was derived by averaging the epochs that had a bpm > 60 and < 120. Values in the heart rate heatmap below 60 bpm were not displayed.

### Statistics

Statistical analysis was performed using custom python scripts or GraphPad Prism software. All data are represented as mean values ± standard deviation unless indicated otherwise. For two-group comparisons, a two-tailed nonparametric Mann–Whitney test was used unless otherwise specified. All analyses except respirophasic inspiratory versus expiratory BCG magnitudes were unpaired. *P* values less than 0.05 were considered significant and are indicated by asterisks as follows: *P < 0.05, **P < 0.01, ***P < 0.001, ****P < 0.0001.

## Supplementary Information

Supplementary Figures.

Supplementary Table S1.

## Data Availability

The data and code that support the findings of this study are available on request from the corresponding author K.R.K. The data are not publicly available because it could compromise research participant privacy/consent.
