# Supplementary material for: Passive longitudinal weight and cardiopulmonary monitoring in the home bed
Source: Sci Rep. 2021 Dec 21;11:24376. doi: 10.1038/s41598-021-03105-1 (PMC8692625; doi:10.1038/s41598-021-03105-1)
Supplement: Supplementary file 1 — Supplementary Figures. [file 41598_2021_3105_MOESM1_ESM.docx]

**
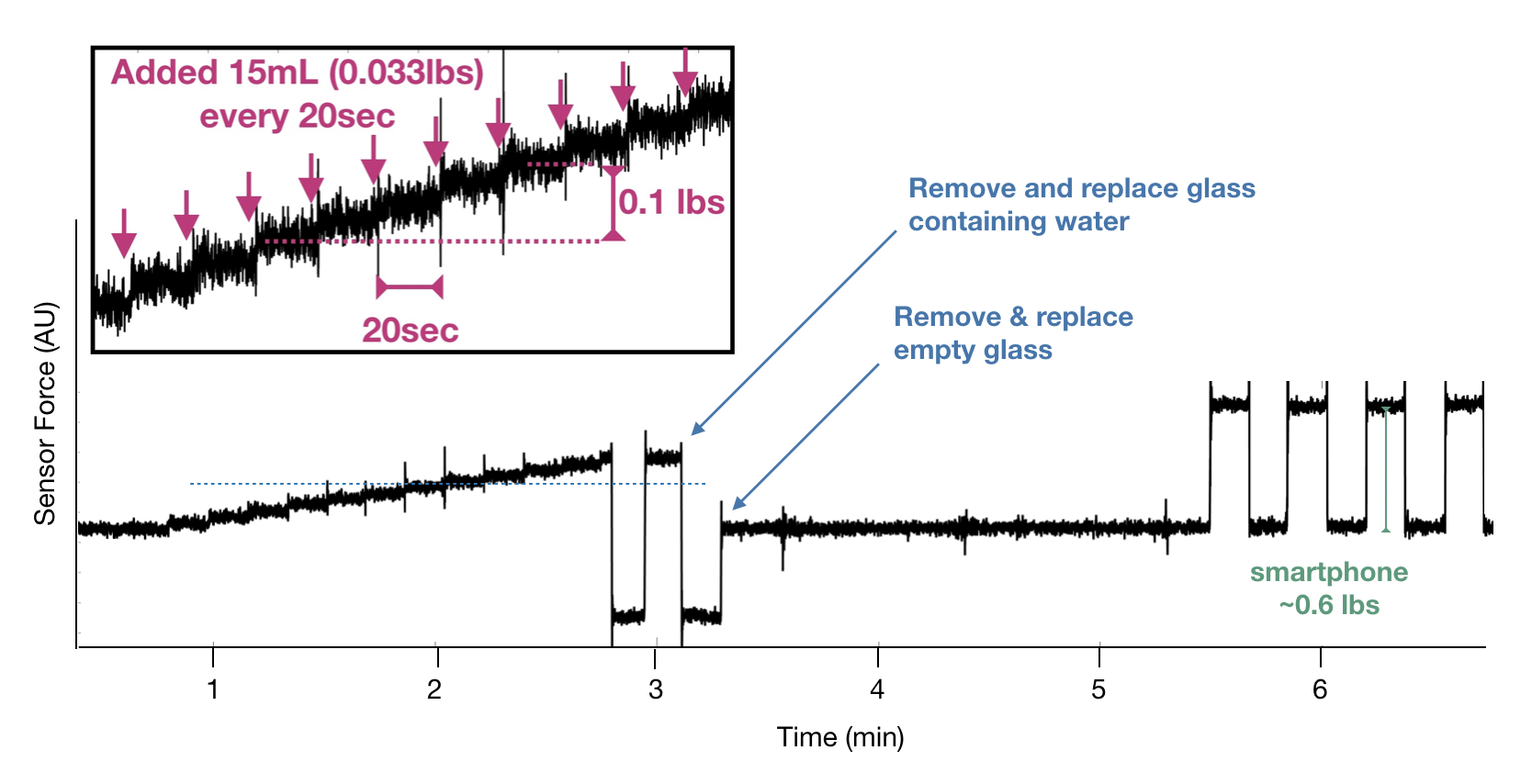
**

**Supplemental Figure S1. BedScales Sensitivity.** To explore the lower limits of sensitivity and resolution, we added 15mL aliquots of water (0.033 lbs) every 20 seconds for a total of 4 minutes (180mL, 0.396 lbs) followed by removal and replacement of the full and empty glass, and removal and placement of a smartphone (~0.6lbs), which are representative of small objects commonly placed on a bed. This revealed the rms noise to be equivalent in magnitude to a ~0.02 lbs object. For comparison, the limit of resolution of conventional bathroom scales is typically 0.2 lbs.


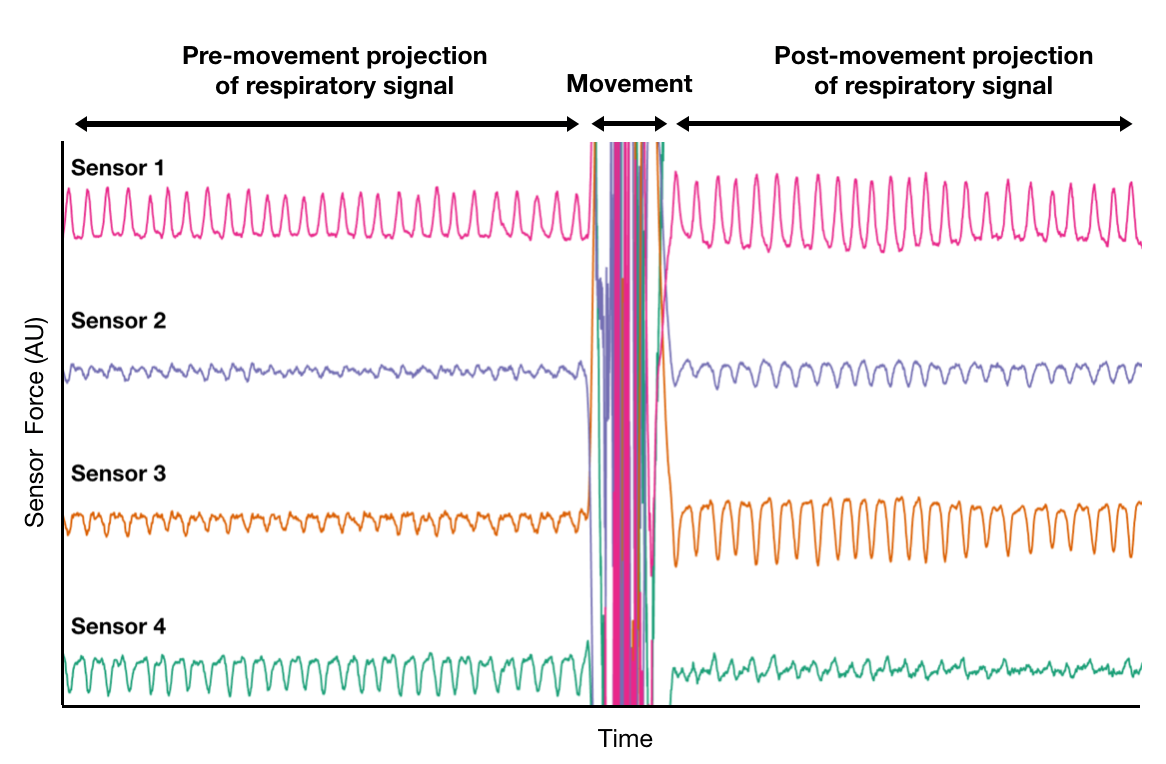


**Supplemental Figure S2. Patients behave like respiratory point sources.** Respiratory signal from the BedScales sensors beneath each of 4 bed legs during sleep. The subject, who is resting still at the beginning of the record, changes position in bed in the middle and returns to resting still at the end of the record. Notice that the subject - the respiratory source - projects to each BedScales sensor with a consistent relative magnitude, which changes after the movement. This allows the subject to be viewed as a respiratory point source that makes discrete episodic changes, thus allowing demixing using source separation mathematics as detailed in the methods.
