# Supplementary material for: Passive longitudinal weight and cardiopulmonary monitoring in the home bed
Source: Sci Rep. 2021 Dec 21;11:24376. doi: 10.1038/s41598-021-03105-1 (PMC8692625; doi:10.1038/s41598-021-03105-1)
Supplement: Supplementary file 2 — Supplementary Table S1. [file 41598_2021_3105_MOESM2_ESM.docx]

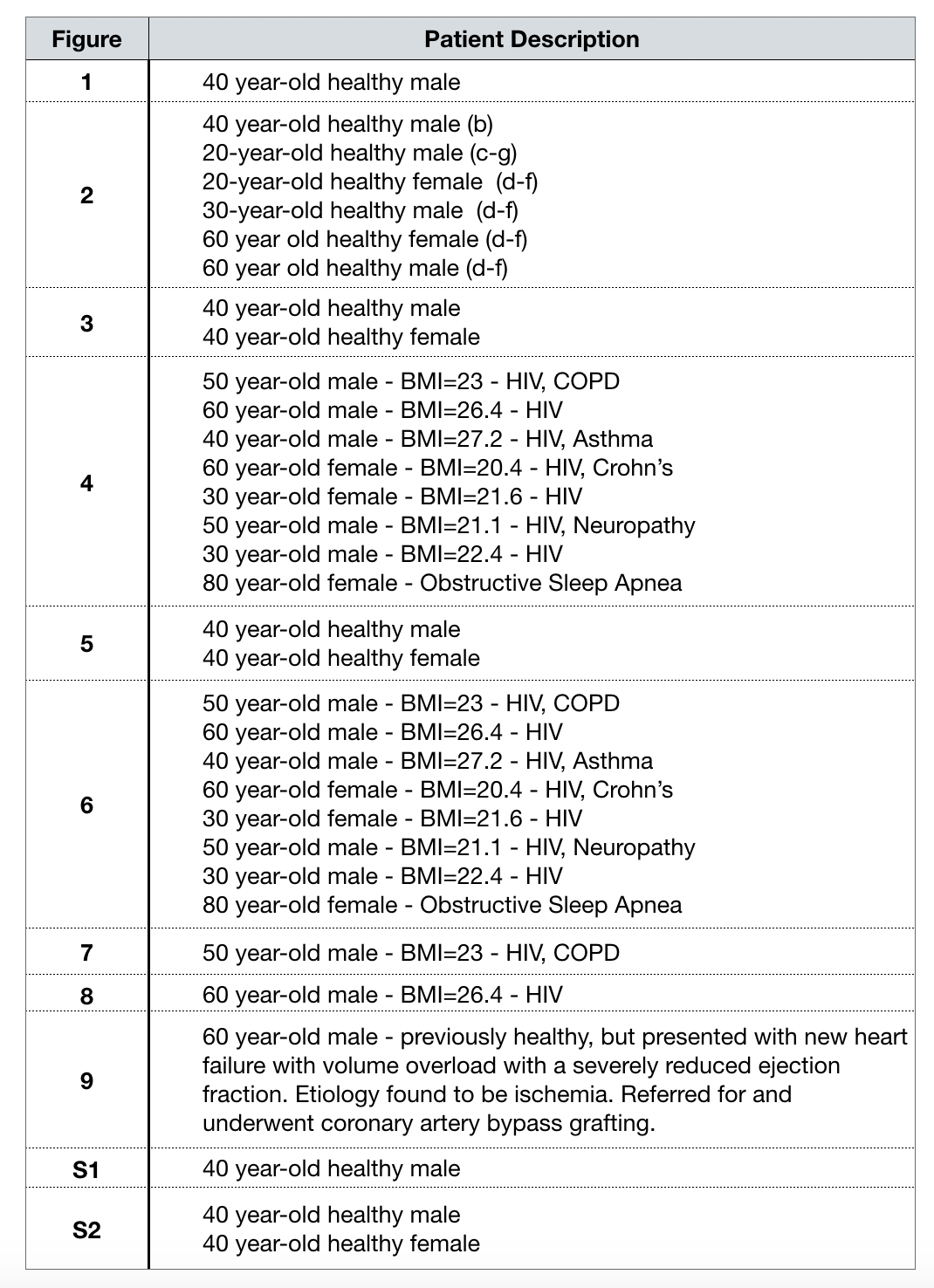


**Supplemental Table S1.** List of human subjects from whom BedScales validation and feasibility studies were collected. Ages are rounded to nearest multiple of 10 to preserve deidentification.
